# A case of fatal multidrug intoxication involving flualprazolam: distribution in body fluids and solid tissues

**DOI:** 10.1007/s11419-021-00591-w

**Published:** 2021-08-11

**Authors:** Arianna Giorgetti, Michaela J. Sommer, Maurice Wilde, Markus Große Perdekamp, Volker Auwärter

**Affiliations:** 1 DIMEC, Department of Medical and Surgical Sciences, University of Bologna, 40126, Bologna Italy; 2 Institute of Forensic Medicine, Medical Center–University of Freiburg, 79104 Freiburg, Germany; 3 Faculty of Medicine, University of Freiburg, 79104 Freiburg, Germany; 4 Hermann Staudinger Graduate School, University of Freiburg, 79104 Freiburg, Germany

**Keywords:** Flualprazolam, Novel psychoactive substance (NPS), Designer benzodiazepine, Triazolobenzodiazepine, Standard addition method, LC–MS/MS

## Abstract

**Purpose:**

Designer benzodiazepines (DBZDs) increasingly emerged on the novel psychoactive substance (NPS) market in the last few years. They are usually sold as readily available alternatives to prescription benzodiazepines (BZDs) or added to counterfeit medicines. BZDs are generally considered relatively safe drugs due to the low risk of serious acute adverse effects in mono-intoxication, though e.g., alprazolam seems to display an elevated risk of respiratory depression. Here we report on a fatal intoxication involving the novel DBZD flualprazolam.

**Methods:**

A complete postmortem examination was performed. General unknown screenings and analysis of drugs of abuse were performed on postmortem samples by immunoassay, gas chromatography–mass spectrometry and liquid chromatography–mass spectrometry. The standard addition method was employed to quantify flualprazolam in postmortem blood and tissues. Finally, a toxicological significance score (TSS) was assigned.

**Results:**

Flualprazolam was detected in heart serum (25.4 ng/mL) and peripheral blood (21.9 ng/mL) as well as in urine, stomach contents, brain, liver and kidney (65.2–323 ng/g). The cause of death was deemed as central nervous system (CNS) and respiratory depression with agonal aspiration of stomach contents, in the setting of a multiple drug intake. Given the concentration levels of the co-consumed CNS depressants, the contribution of flualprazolam to the death was considered likely (TSS of 3).

**Conclusions:**

Our results support that highly potent DBZDs like flualprazolam carry an elevated risk for unintended toxicity, especially in association with other CNS depressants. A multidisciplinary evaluation of fatalities remains mandatory, especially when pharmacological/toxicological data on intoxicating compounds are lacking. To our knowledge this is the first report of flualprazolam concentrations in solid tissues in human.

## Introduction

The term ‘designer benzodiazepines’ (DBZDs) or ‘novel benzodiazepines’ usually refers to a class of new psychoactive substances (NPS) designed as alternative to ‘prescription-only’ or controlled benzodiazepines (BZDs). The majority has never been licensed as a medical drug or has been approved only for a limited period of time or in certain regions. In analogy to other designer drugs, DBZDs are commonly sold without effective restriction on the ‘legal high’ drug market at relatively cheap prices, and structural modifications have been introduced to circumvent national narcotics laws [1–3].

The European Monitoring Centre for Drugs and Drug Addiction (EMCDDA) is actually monitoring 30 DBZDs, 21 of which have been offered on the NPS market only since 2015 [1]. The EMCDDA also identified a significant increase in the amount of seizures of material containing DBZDs, with close to 3500 seizures in 2017, as compared to nearly 4700 seizures in 2018 [1]. Accordingly, within the STRIDA project which monitors the occurrence of NPS related intoxications in Sweden, an increased rate of positive urine sample for DBZDs has been reported (from 4% in 2012 to 19% in 2015) [4].

The pharmacological profile and the effects of DBZDs are supposed to be similar to those of therapeutically used BZDs, which are positive allosteric modulators of GABA_A_ receptors. In contrast to other central nervous system (CNS) depressants like barbiturates,  BZDs are known to be relatively safe drugs. Indeed, they are rarely found as the cause of death in mono-intoxications, although the combination with other drugs and medications, e.g., opioids or alcohol, can easily lead to death [5–7].

As there is currently no recognized medical indication, DBZDs are assumed to be used as a substitute for prescription-only BZDs, as a ‘self-medication’ in the setting of other drug intake, in particular with stimulants and hallucinogens, or as self-treatment of sleep or anxiety disorders. Some DBZDs are also found as counterfeit products of medically approved BDZs, a phenomenon that has particularly been described for alprazolam and diazepam [8]. Finally, they could be consumed to seek a recreational effect. Up to now, data regarding safety, toxicity and potency of these compounds are widely missing. Therefore, they are considered to represent a potential public health problem with a high risk for unintended intoxication or death and development of addiction [9].

**Fig. 1:**
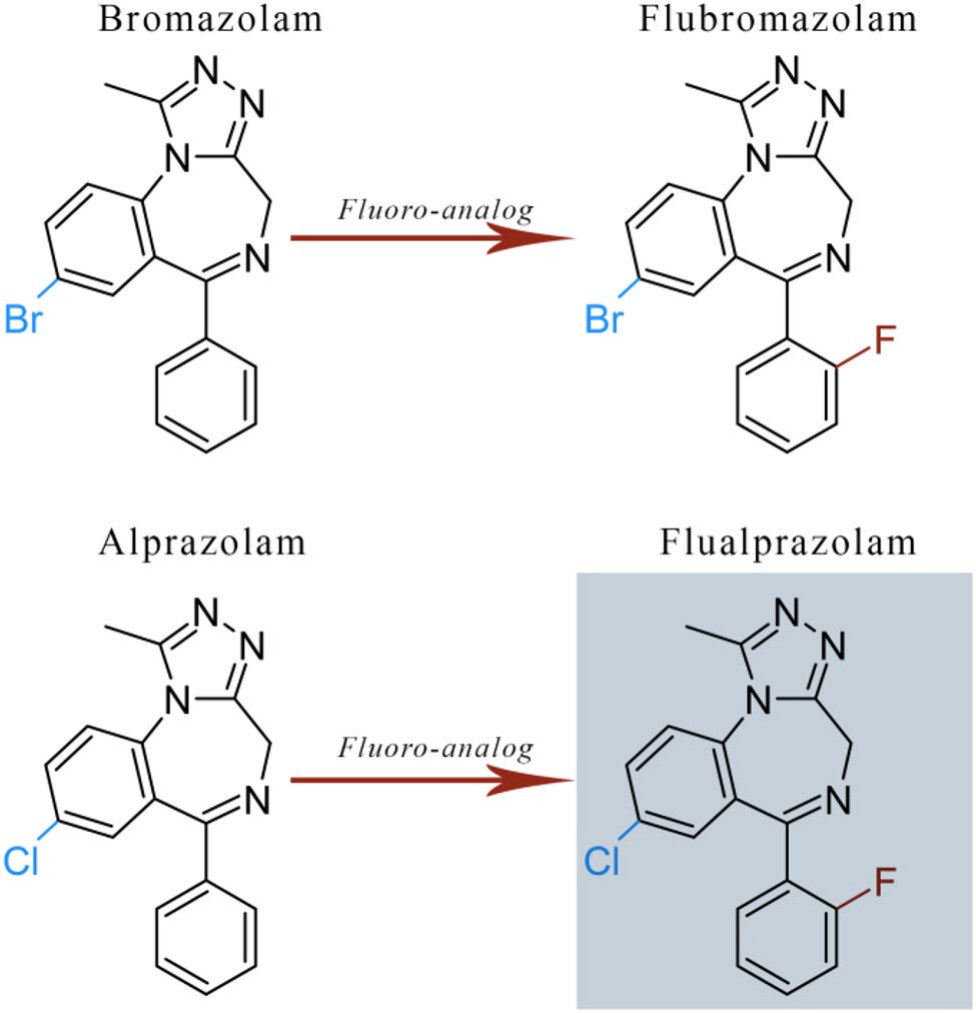
Chemical structures and semi-systematic names of flualprazolam and related benzodiazepines/designer benzodiazepines

Flualprazolam is the semi-systematic name for 8-chloro-6-(2-fluorophenyl)-1-methyl-4*H*-benzo[f][1,2,4]triazolo[4,3-a][1,4]diazepine and can be regarded as the 2-fluoro derivative of alprazolam. It is a triazolo  BZD structurally related to the DBZD flubromazolam, with the replacement of the chlorine by a bromine atom in the 8-position of the triazolo benzodiazepine moiety (Fig. 1). It appeared on the NPS market for the first time in 2017 [10] and has been seized in the form of tablets, ‘blotters’ or as a ‘research chemical’ (powder form) in many European countries between 2018 and 2019 [1]. Moreover, it has recently been linked to a number of intoxications and deaths in Northern Europe and in the United States [11–15].

Very little is known regarding pharmacokinetics and pharmacodynamics of flualprazolam [16], although Wagmann et al. [17] studied the in vivo and in vitro metabolism of flualprazolam and identified its main metabolites. Triazolo analogs (e.g., flubromazolam) are generally more potent than the corresponding 1,4-benzodiazepines and exhibit longer elimination half-lives. They were shown to have the highest GABA_A_ receptor binding affinities among DBZDs [18].

## Case history

In February 2019, a 21-year-old male with a history of drug abuse, including hypnotics and occasional BZD consumption, was found lying unresponsive on his bed, with a blueish discoloration of the lips, at 1:00 am in the night. In the same room, two other men were hosted, one of whom admitted that they had consumed ‘speed’. The patient was immediately brought to the hospital and resuscitated. Despite cardiopulmonary resuscitation, he was declared dead approximately 2 h after the retrieval efforts. Serum samples taken at the hospital and postmortem samples were available for analysis.

## Materials and methods

### Reference materials and chemicals

The reference standards (RSs) (bromazepam and flualprazolam) and the internal standards (ISs) (bromazepam-D_4_ and nordazepam-D_5_) were purchased from Cayman Chemicals (Ann Arbor, MI, USA), Lipomed GmbH (Weil am Rhein, Germany) and LGC standards GmbH (Wesel, Germany). Hydroxyflualprazolam was not commercially available. The reference spectrum of hydroxyflualprazolam was generated by incubation with pooled human liver microsomes and used as positive control for the detection of this analyte (*m/z* 343.1—> 325.3, 343.1—> 298.2). Methanol (HiPerSolv CHROMANORM^®^) and acetonitrile (ACN) (HiPerSolv CHROMANORM^®^) were purchased from VWR Chemicals (Darmstadt, Germany); formic acid (> 98% p.a.) from Carl Roth GmbH (Karlsruhe, Germany); ammonium formate solution (10 M) from Sigma-Aldrich (Steinheim, Germany); 1-chlorobutane (LiChrosolv^®^) from Merck (Darmstadt, Germany); β-glucuronidase solution (from *E.coli* K12) from Roche Diagnostics GmbH (Mannheim, Germany). Deionized water was prepared using a Medica^®^ Pro deionizer from ELGA (Celle, Germany). Borate buffer (38.9 g/L boric acid, 47 g/L potassium chloride and 39.2 g/L sodium carbonate in deionized water, pH adjusted to 9) and phosphate buffer (13.6 g/L potassium dihydrogenphosphate in deionized water, pH adjusted to 6) were freshly prepared prior to use. Mobile phase A (0.1% v/v formic acid, 1 mM ammonium formate in water) and mobile phase B (0.1% v/v formic acid in methanol) were freshly prepared prior to analysis.

### Postmortem examination and sampling

A postmortem examination was performed 2 days after the death. Samples of tissues were fixed in formalin, dyed with standard hematoxylin and eosin (H&E) and then analyzed by optical microscope. A serum sample collected at admission to the emergency department was requested and submitted to toxicological analyses.

### Toxicological analyses

The screening and tentative quantification of classical drugs of abuse and ‘general unknown screenings’ were performed by CEDIA and DRI^®^ immunoassays, gas chromatography (GC) with flame-ionization detection (FID), GC–mass spectrometry (GC–MS), and liquid chromatography (LC) with tandem mass spectrometry (LC–MS/MS) using validated methods accredited under ISO/IEC 17,025 for forensic purposes. Antemortem serum samples and urine samples (after enzymatic hydrolysis by ß-glucuronidase) were analyzed by means of LC–MS/MS using a previously published method for DBZDs after update and revalidation [19, 20].

The quantification of flualprazolam in fluids and tissues samples taken at the postmortem examination was done by a standard addition approach to overcome the strong matrix effects expected in such material. For postmortem blood, aliquots of 100 µL for each specimen containing an unknown amount of the analyte of interest were spiked with deuterated IS solution (nordazepam-*D*_5_) and varying volumes of flualprazolam RS solution. A six-point calibration curve, including a zero sample (only blood with IS) was prepared in this manner, with the following final concentrations: 0, 1, 5, 10, 25, 50 ng/mL. The samples were worked up by a liquid-liquid extraction procedure described by Moosmann et al. [19]. In brief, 900 µL of borate buffer and 1 mL 1-chlorobutane were added to the samples. The samples were mixed in an overhead shaker and centrifuged at 4000 rpm for 15 min. The supernatant was transferred into a glass vial, evaporated to dryness under a gentle stream of nitrogen (40 °C) and reconstituted in 100 µL LC–MS mobile phase.

For the analysis of tissue samples and stomach contents, samples were minced with clean surgical scissors. Then, 0.5 g of the material was placed in a 1.5 mL plastic tube with a cap containing 1 mL of ACN. Five stainless steel beads were added to the mixture; the tube was capped, put into a homogenizer (Beads Crusher lT-12; TAITEC, Koshigaya, Japan), and vigorously shaken at 3200 rpm for 30 s. The homogenates of brain, kidney and liver were further diluted with 3 mL ACN (resulting in 0.5 g in 4 mL ACN); the homogenate of the stomach contents was further diluted with 11 mL (resulting in 0.5 g in 12 mL ACN). The homogenized samples were stored at − 20 °C for at least 24 h prior to extraction. For the standard addition procedure of tissue homogenates, 100 µL supernatant of the homogenized specimen were equally aliquoted, spiked with RS and/or IS and extracted using the extraction method as described above. The resulting tissue calibrators were: 0, 4, 20, 40, 100, 200 ng/g for brain, liver and kidney and 0, 12, 60, 120, 300, 600 ng/mL for stomach contents. For intraday repeatability, the described procedure was performed 5 times for each specimen.

### Toxicological significance score

According to the literature [21], a toxicological significance score (TSS) was assigned. The TSS was developed to support the risk assessment of NPS. It assesses and classifies the role of the NPS in fatalities. It requires consideration of many factors, such as the presence, nature and concentration of the NPS as well as other drugs present, circumstances of death and further factors. TSS can range from ‘1’, i.e., ‘alternative cause of death’, to ‘3’, i.e., ‘NPS is considered likely to have contributed to toxicity/death, even in presence of other drugs’.

## Results

### Postmortem examination

**Fig. 2:**
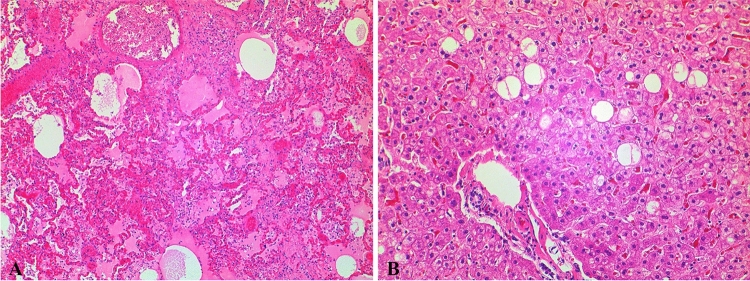
Histology of lung tissue (**A**), showing severe hemorrhagic edema (× 4) and of liver tissue (**B**), displaying microvesicular steatosis (× 20)

The deceased was overweight and with shaved head (thus, no hair sample was available for analysis). Signs of cardiopulmonary resuscitation and a smell of ethanol were noted. The heart showed a mild dilatation of the right chambers, atherosclerosis, with a noncritical stenosis (> 50%) at the left descending coronary artery, and patency of the foramen ovale. In the trachea, a film of brown liquid was seen, mixed with small food particles, consistent with aspiration of stomach contents (approx. 200 mL). The lungs (overall weight: 1170 g) were congested and edematous, with signs of chronic sclerosis. The brain also displayed severe edema (1735 g), with herniation of the cerebellar tonsils. The liver was enlarged (2632 g), fatty and congested. Microscopically, hemorrhagic edema were seen in both lungs (Fig. 2A), with thickened septa and intra-alveolar desquamation cells, as in the cases of alveolar damages. The liver showed a microvesicular steatosis (Fig. 2B).

### Toxicological analyses

Immunochemical tests gave positive results for benzodiazepines, amphetamine, methamphetamine, and cannabinoids in antemortem serum from the hospital and postmortem urine. A positive result for opiates was only observed for urine. Ethanol was found positive in both femoral blood and urine samples, with concentrations of 0.95 and 1.90 g/L, respectively. In postmortem femoral blood, flualprazolam (21.9 ng/mL), its metabolite hydroxyflualprazolam, bromazepam and flumazenil as well as tilidine and nortilidine were detected by LC–MS/MS. Urine analysis confirmed the benzodiazepine findings in the femoral blood sample [flualprazolam (97 ng/mL), hydroxyflualprazolam, bromazepam (4.6 ng/mL), 3-hydroxybromazepam (14 ng/mL) in urine]. Furthermore, amphetamine, tilidine, nortilidine, flumazenil and naloxone were detected in the urine sample. Screening analyses in urine for other NPS (synthetic cannabinoids, designer stimulants/hallucinogens and designer opioids) were negative. The creatinine level of the urine sample was 34 mg/dL.

The serum sample from the hospital tested positive for amphetamine (28 ng/mL), tilidine (86 ng/mL), its active metabolite nortilidine (approx. 250 ng/mL) and the opioid antidote naloxone. γ-Hydroxybutyrate (GHB) level (< 2.5 mg/L) was unremarkable. Blood ethanol was 0.94 g/L. The LC–MS/MS analysis for BZDs and DBZDs resulted in the identification of flualprazolam (37 ng/mL), hydroxyflualprazolam and bromazepam (9.4 ng/mL). A summary of the toxicological findings of the antemortem serum is given in Table 1. The results of the remaining postmortem specimens were consistent with the postmortem findings in blood and urine. In vitreous humor, in addition to flualprazolam and bromazepam, amphetamine as well as tilidine/nortilidine, naloxone and flumazenil were detected. Analyses of the stomach contents revealed the presence of tilidine, flualprazolam and alcohol (1.56 g/kg). Concentrations and intraday repeatability for determination of flualprazolam in postmortem specimens using standard addition calibration are listed in Table 2. Table 3 shows the standard addition calibration equations for flualprazolam in the different specimens.

**Table 1:** Tentative quantitative analyses of the serum  obtained antemortem  at the clinic during resuscitation

Substance	Concentration (ng/mL, mg/L for GHB)
Flualprazolam	37
Hydroxyflualprazolam	n.q
Bromazepam	9.4
GHB	< 2.5
THC-COOH	< 5
Amphetamine	28
Tilidine	86
Nortilidine	250^a^
Naloxone	4.9
Flumazenil	n.q

*GHB* γ-hydroxybutyric acid, *THC-COOH* 11-nor-9-carboxy-Δ^9^-tetrahydrocannabinol, *n.q*. not quantified

^a^Extrapolated (above the calibration range)

**Table 2:** Flualprazolam concentrations in postmortem samples determined using the standard addition method (urine was measured with a validated method)

Specimen	Concentration^a^	Repeatability (% RSD)
Heart serum	25.4 ± 0.8 ng/mL	3.18
Femoral blood	21.9 ± 0.8 ng/mL	3.69
Brain	65.2 ± 9.4 ng/g	14.4
Stomach contents	323 ± 36 ng/g	11.1
Liver	97.7 ± 6.4 ng/g	6.50
Kidney	73.1 ± 12.7 ng/g	17.4
Urine	97 ng/mL	

*RSD* relative standard deviation

^a^Concentrations are given as mean ± standard deviation (SD) obtained by five intraday replicates

**Table 3:** Standard addition calibration equations for flualprazolam in body fluids and solid tissues of the deceased

Specimen	Equation	Correlation coefficient (*r*)
Heart serum	*y* = 14.6 *x* + 372	0.995
Femoral blood	*y* = 17.6 *x* + 387	0.986
Brain	*y* = 17.7 *x* + 145	0.990
Stomach content	*y* = 19.0 *x* + 252	0.977
Liver	*y* = 24.7 *x* + 306	0.990
Kidney	*y* = 22.4 *x* + 201	0.989

*y* area ratio of flualprazolam to internal standard, *x* concentration of flualprazolam

## Discussion

BZDs mediate sedative, amnesic, anticonvulsive, muscle relaxant and anxiolytic effects. Acute intoxications related to DBZDs usually present with disorientation, lethargy, confusion, and slurry speech, and paradoxical agitation, tremor and tachycardia were also reported [4, 22–25].

The non-fluorinated analog of flualprazolam, alprazolam, is one of the most common BZDs in fatal polydrug intoxications [26, 27], often due to the amplification of the respiratory depressive effects induced by co-consumed compounds [28]. Toxic serum ranges are described from 100 to 400 ng/mL [28–31], but widely overlap with serum ranges reached under therapeutic use [31–33]. For alprazolam overdoses, mechanical ventilation, intensive care unit admission and flumazenil administration are more commonly required as compared to other BZDs, though not being more frequently associated with coma [34]. The general risk of severe CNS depression was also confirmed by cases of mono-intoxications [6, 35, 36]. On the other hand, alprazolam is one of the most frequently prescribed BZDs [37, 38] and carries a high risk for misuse and diversion, especially in individuals with substance use disorders [39]. Indeed, together with clonazepam and diazepam, alprazolam is one of the most commonly detected BZDs in opioid users and acute heroin intoxications [40]. Thus, it is possible that the perceived risks associated with alprazolam intoxication are in fact due to the circumstances of use rather than to an elevated intrinsic toxicity. Other triazolo BZDs, such as triazolam, the chloro-analog of flualprazolam, have been linked to fatal intoxications [41–45]. In serum, potentially toxic concentrations of triazolam are described starting at about 40 ng/mL [31]. Generally, deaths caused by DBZDs alone are rare [4]. Deep coma and acute respiratory failure were reported in a 27-years-old man who ingested 3 mg of flubromazolam (59 ng/mL in serum). However, the patient survived, thanks to mechanical ventilation, and norepinephrine and flumazenil administration [46].

Despite the lack of safety and toxicity tests, given its structure and similarity to flubromazolam, clinical and unwanted/unexpected effects of flualprazolam are to be expected at lower concentrations than with alprazolam. NPS users reported long lasting and strong effects at low doses, as reported e.g., by Huppertz et al. [22] for 0.5 mg of flubromazolam. According to TripSit FactSheets [47], common doses of flualprazoam are similar to those of flubromazolam and were reported at 0.25–0.5 mg [10, 24]. According to an article posted at the social news aggregation provider Reddit [48], such a dose could be considered equivalent to about 2 mg of alprazolam, suggesting a 4- to 8-fold higher potency. The onset of effects is reported at 10–30 min after ingestion, with a duration of 6–14 h, similar to e.g., clonazolam or fluclotizolam [10, 16, 49].

Very recently, flualprazolam has been detected in forensic cases in several countries. Flualprazolam was rarely the only substance of relevance for impairment; in the majority of cases, other drugs were detected as well. In Norway, 10 survived cases of intoxication (median level in blood 8 ng/mL, 75th percentile 25 ng/mL, maximum 56 ng/mL) have been reported [13]. Further, 33 postmortem samples were reported from Sweden and Finland (median 18 ng/g in femoral blood, range 3.0–68 ng/g) [12] and 197 postmortem and driving under the influence of drugs (DRUID) cases from the United States [11]. In the latter, average concentrations in postmortem blood (*n* = 167) and blood from DRUID cases (*n* = 22) were 20 ± 63 ng/mL and 22 ± 18 ng/mL, respectively.

In the here reported case, an agonal aspiration of stomach contents occurred. In a young, otherwise healthy person (with only minor cardiac findings bearing no clinically relevant functional consequences) and in the absence of other causes, this could be explained only as a consequence of a state of reduced consciousness. This represents a strong hint for a drug-mediated CNS depression. The severe cerebral edema found at the postmortem examination is a sign of long-lasting cerebral hypoxia, thus confirming a prolonged agony time, consistent with a comatose state. The lungs displayed severe acute hemorrhagic edema, which are an unspecific and common finding in acute drug intoxications, particularly in cases of opiate/opioid overdose. The pathogenesis of hemorrhagic edema following heroin intoxication is still not well understood, and possible mechanisms include CNS depression, increase of the permeability of pulmonary capillary, depressed myocardial function or direct toxic effect on capillaries [50]. Intra-alveolar hemorrhages and severe edema were also described in cases of alprazolam intoxications [29, 35] and are consistent with a respiratory depression exerted by DBZDs. In addition to flualprazolam, which was found at higher concentrations than the median concentration of postmortem cases reported so far [11, 12], several illicit and prescription drugs were detected. Bromazepam is used as an antianxiety medication in doses ranging from 3–12 mg. Even though multiple factors have to be considered when evaluating the role of BZDs in fatalities, including the pattern of use/abuse of the victim, which could result in tolerance [6], the bromazepam concentration (Table 1) was below the usual therapeutic serum levels (80–200 ng/mL, with toxicity described at concentrations above 300 ng/mL [31]). The deceased was allegedly a  BZD user. Since counterfeit forms of  BZDs available on the Internet might contain unwanted designer compounds [1], it is not possible to establish if the intake of flualprazolam was intentional or not. However, the friends stated that they were consuming ‘speed’, which might point to an unintended uptake of flualprazolam in powder form instead of amphetamine. On the other hand, DBZDs are often co-consumed with CNS stimulants to counterbalance their effects [4]. Here, the circumstantial data of ‘speed’ intake was analytically confirmed (Table 1), but given the low blood concentration, amphetamine use shortly before death is unlikely. Although in general amphetamine does have effects on the cardiovascular function and can induce arrhythmia, a contributory role seems unlikely in this case.

Tilidine and its main metabolite nortilidine were found in slightly elevated ranges (Table 1) when compared to concentrations usually found after therapeutic use (50–300 ng/mL for tilidine, around 200 ng/mL for nortilidine [31, 51]). In Germany, tilidine is used as an opioid analgesic, mostly in combination with naloxone for oral intake [52]. While tilidine itself is a scheduled substance, retarded dosage forms with the fixed combination of naloxone and tilidine are excluded from the German Narcotics Law [51]. Cases of multidrug intoxication including tilidine have been reported, with respiratory depression resulting in pulmonary edema as the main autopsy finding [53, 54]. The detection of the drug in the gastric contents points to a probable oral consumption, whereas the absence of naloxone (confirmed only in serum) in the same specimen speaks against a co-ingestion of the drugs and suggests that naloxone was administered as an antidote in the hospital setting instead. The administration of naloxone and flumazenil (antidote for BZDs) is common practice during resuscitation in cases of suspected drug intoxication [55].

A history of alcohol misuse is likely in the present case, since liver steatosis was seen. In the blood sample taken in the hospital, the blood alcohol concentration (BAC) was 0.94 g/L and in femoral blood almost identical, which is consistent with ineffective resuscitation. Therefore, at the time of death a moderate CNS depressant ethanol effect can be assumed. Given the ratio of 2.0 between urinary alcohol concentrations and BAC, as in the post-peak phase, BAC could have been higher in the agonal phase preceding death [56]. Respiratory arrest and coma induced by alcohol alone typically occur at blood levels above approx. 3.5 g/L [31, 57, 58]. Nevertheless, CNS depressant effects of ethanol probably significantly contributed to the fatal outcome.

Given the concentrations of ethanol and tilidine/nortilidine, the signs of respiratory depression found at the postmortem examination and the high potency and levels of flualprazolam, this DBZD appears to be a crucial factor in this death case. Therefore, a TSS of 3 was assigned in the setting of multiple drug and alcohol intake with synergistic CNS depressant effects.

The concentrations of flualprazolam were similar to those reported in drug-related deaths due to other triazolo BZDs such as triazolam [45], and below those usually related to toxic effects of alprazolam [31]. This would be in line with a much higher potency of flualprazolam as compared to its analog alprazolam, as it can be assumed following known structure-activity relationships for BZDs [59].

No data on stability, red blood cell/plasma partition, or postmortem redistribution are currently available for flualprazolam. The lower flualprazolam level measured in peripheral blood when compared to heart serum points towards a blood to serum ratio of about 0.86 (Table 2) which would be close to the ratio determined for e.g., diazepam (0.71) [60]. The similar concentrations measured in the clinical serum sample and in the postmortem heart serum do not suggest relevant postmortem redistribution. However, given the relatively high concentrations measured in the stomach contents and in liver tissue (Table 2), it seems possible that postmortem heart levels slightly rose due to a passive diffusion from the surrounding tissues. For alprazolam, Wolf et al. [32] stated that no postmortem redistribution occurred.

An oral intake of flualprazolam is likely in this case, given the high levels detected in stomach contents. Concentrations were high not only in the stomach, which suggests recent intake and incomplete absorption, but also in liver and kidney tissues and urine (Table 2). Therefore, an intake of several flualprazolam doses at different times could have occurred; the possibility that an (additional) uptake by insufflation in the case of confusing amphetamine and flualprazolam powder cannot be excluded. Among the solid tissues, higher concentrations were detected in the liver, as already shown in a fatal alprazolam intoxication [35]. The detection of flualprazolam in brain tissue supports CNS effects at the moment of death and is an expected finding due to the lipophilicity of the compound. To our knowledge, this is the first report to present the concentrations of flualprazolam in solid tissue specimens, while flualprazolam concentrations in numerous human blood samples have been reported [11, 12].

## Conclusions

We report a case of death in which the DBZD flualprazolam was involved and likely contributed to death (TSS = 3) in the setting of multidrug intake. Our results support the hypothesis of an extraordinarily high potency of flualprazolam and its potential for severe CNS and respiratory depression. Given the potential for fatal intoxications, particularly when combined with other CNS depressants, their easy availability at relatively low prices and the elevated risk of unintended overdoses, DBZDs deserve increased attention. The present case further confirms that, especially when no data is available regarding toxic doses or concentration levels, a careful and multidisciplinary evaluation of all data is necessary to assess the contributory role of the substance in death cases.
